# Cumulative Ecological Risk and Problem Behaviors Among Adolescents in Secondary Vocational Schools: The Mediating Roles of Core Self-Evaluation and Basic Psychological Need Satisfaction

**DOI:** 10.3389/fpubh.2021.591614

**Published:** 2021-02-09

**Authors:** Peizhen Sun, Yudi Sun, Delan Fang, Hongyan Jiang, Mengjie Pan

**Affiliations:** ^1^Department of Psychology, School of Education Science, Jiangsu Normal University, Xuzhou, China; ^2^School of Management, China University of Mining and Technology, Xuzhou, China; ^3^School of Preschool and Special Education, Xuzhou Kindergarten Teachers College, Xuzhou, China; ^4^International College, Jiangsu Normal University, Xuzhou, China

**Keywords:** cumulative ecological risk, problem behaviors, core self-evaluation, basic psychological need satisfaction, adolescents

## Abstract

Based on the Cumulative Risk Model, a single risk factor cannot play a decisive role, but the cumulative ecological risks may have complex superposition effects on adolescents' problem behaviors. However, although many studies have investigated the specific influences of single external risk factors on problem behaviors, the effect of cumulative ecological risk on problem behaviors and especially the underlying mechanisms therein have been under-investigated. Therefore, this study aimed to explore the effect of cumulative ecological risk on adolescents' problem behaviors, and the mediating effects of core self-evaluation and basic psychological needs satisfaction therein. To achieve this, 1,080 adolescents in secondary vocational schools were surveyed with the questionnaires of cumulative ecological risk, basic psychological need satisfaction, core self-evaluation, externalizing and internalizing problem behaviors. Results showed that: (a) cumulative ecological risk was positively related with both internalizing problem behavior and externalizing problem behavior; (b) core self-evaluation and basic psychological need satisfaction not only respectively but also sequentially mediated the relationship between cumulative ecological risk and two sorts of problem behaviors. These results provide some pivotal implications for the precaution and intervention of the adolescent problem behaviors.

## Introduction

In recent years, the Chinese government has spared no effort to develop the vocational education in order to satisfy the need for the vocational talents (1). The secondary vocational education begins to hold a more and more important position in the entire education system. On this background, the health and behavior problem of students in secondary vocational schools has become a hot topic in educational world and has attracted the attention of researchers as well. In China, vocational school students are mainly from the junior middle school graduates who flunk the entrance examination for regular high school because of unsatisfied academic performance (2). Compared with regular high school students, vocational students in China are ordinarily characterized by heterogeneous educational levels. Insufficiency in educational attainment and coping skills may expose them to more risky factors that they fail to handle with (3). In addition, secondary vocational schools have less competitive teaching faculties, lower education levels and poorer conditions (4). The unique internal and external environment faced by secondary vocational students makes them more prone to health and problem behaviors.

Problem behaviors are abnormal behaviors that have detrimental effects on individuals' physical and mental development (5). They include externalizing and internalizing behaviors. Internalizing problem behaviors are emotion-oriented problem behaviors involving negative emotions such as anxiety, depression, and withdrawal (6). Externalizing problem behaviors are behavior-oriented and include aggression and delinquency (7). Previous researches of predecessors have investigated the specific influences of single external risk factors on problem behaviors (8, 9). But in real life, people are often influenced by multiple risk sources, including peers, family, school, and community. At the meantime, according to the Cumulative Risk Model (10), a single risk factor cannot play a decisive role in problem behaviors. When a person confronts more than one risk factor, multiple risks will have complex interaction and superposition effects. In addition, the more risk factors interact, the stronger their negative impact will be on the person, which promotes problem behaviors (11). Therefore, it is essential to investigate the influence of cumulative risk factors on problem behaviors. Based on the above discussion, this study attempts to shed light on the impact of cumulative ecological risks on problem behaviors among secondary vocational school adolescents and further unpack the black box of their mediation mechanism.

### Cumulative Ecological Risk and Problem Behaviors

Cumulative ecological risk refers to risk sources coming from multiple domains, for instance, peers, family, school, and community, in a person's living environment. It may have an unfavorable influence over individual development (10). According to the Cumulative Risk Model, such risk factors do not operate independently in exerting harmful influences on adolescent behaviors; the behaviors are shaped by a synergistic effect from multiple interrelated factors (10, 12). Furthermore, empirical research has proved that the overlapping and amassing of ecological risk is one of the most important factors which could influence internalizing and externalizing problem behaviors (13). There are considerable empirical proofs for this standpoint that cumulative ecological risk is positively correlated with problem behaviors, for instance internet addiction (14), alcohol use (15), aggression (16), and depression (17). Guided by aforementioned researches, the first hypothesis was proposed:

**Hypothesis 1:** Cumulative ecological risk is positively related with both internalizing problem behavior and externalizing problem behavior.

Although studies have examined the direct association between cumulative ecological risk and adolescent problem behaviors, the underlying mechanism remains unclear. In light of this, we introduce two mediating variables: basic psychological need satisfaction and core self-evaluation to broaden and reinforce the comprehension of the relationship between cumulative ecological risk and adolescent problem behaviors.

### The Mediating Effect of Basic Psychological Need Satisfaction

Basic psychological need refers to innate organismic necessities and psychological nutriments (18). Based on the self-determination theory, it includes three types, namely, the needs for autonomy (i.e., the desire for self-organize experience and actions), the needs for competence (i.e., the feelings of efficacy to pursue goals and perform tasks) and the needs for relatedness (i.e., the desire for being part of a group or connected to significant others) (19). The satisfaction of basic psychological need is essential for sustained psychological development, integrity and well-being (20). Abundant empirical studies have shown that basic psychological need satisfaction serves as a mediating role not only between a favorable environment (e.g., parents and teachers' supportive behavior, positive parenting) and positive development (e.g., high happiness, self-esteem, or academic achievement) (21, 22), but also between an adverse environment (e.g., need frustration, high pressure, controlling parenting) and undesirable consequences (e.g., malfunctioning, anxiety, depression, behavioral problems) (22, 23).

This study contends that basic psychological need satisfaction could mediate the link between cumulative risk and problem behaviors. First, the accumulation of risk factors from multiple fields may hinder the fulfillment of the basic psychological needs of adolescents (13). Self-determination theory (SDT) emphasizes that whether psychological needs are satisfied or not depends upon whether the environment can provide sufficient supportive resources (18). According to this theory, when individuals are surrounded by cumulative ecological risks, they feel that things are out of their control, and they have a weaker sense of belonging to a school, society, or family, and thus their basic psychological needs are severely impeded (22, 23). In terms of empirical evidences, Vansteenkiste et al. (22) indicated that encountering the controlling, critical or rejecting social risky environment may impede individuals' basic psychological need satisfaction. Likewise, Corrales et al. (23) found that severe adversity (accumulated risk factors) adolescents faced would incur unmet basic psychological needs.

Second, lower basic psychological need satisfaction could serve as an interior driving force to internalizing and externalizing problem behaviors. This is because the inability to satisfy these needs interferes with the individual's normal life, which can result in internalizing and externalizing problem behaviors. For instance, Hein et al. argued that once individuals' basic psychological needs are thwarted, they were prone to have more internalizing and externalizing problem behaviors (e.g., anger, bullying behaviors) (24). Yu et al. also found the inhibiting effect of basic psychological needs satisfaction on specific externalizing problem behaviors, which was internet addiction (25). Similarly, prior scholars verified that those who have inadequate basic psychological need satisfaction are more likely to slip into internalizing problem behaviors (e.g., anxiety and depression) in adolescent (26) and adult groups (27) respectively.

In sum, cumulative ecological risk can significantly negatively predict the satisfaction of basic psychological needs, further facilitating adolescent problem behaviors. Therefore, we advance the following hypothesis:

**Hypothesis 2:** Basic psychological need satisfaction is a significant mediator between cumulative ecological risk and problem behaviors.

### The Mediating Effect of Core Self-Evaluation

Core self-evaluation (CSE) is the primary evaluation of one's own capability and worthiness, which are normally defined by four pivotal factors: self-esteem, self-efficacy, neuroticism, and locus of control (28, 29). Studies have shown that single risky factor can exert a negative impact on an individual's core self-evaluation (30, 31). To put it concretely, Song et al. have shown that lack of social support, one of the risk factors, makes it difficult for individuals to objectively evaluate themselves (30). Hence, individuals with low social support tend to underestimate their own abilities. Likewise, French et al. (31) explored the impact of insufficient parent-child interactions (one ecological risk in the family domain) on adolescents' core self-evaluation. When daily interactions between children and parents are reduced, adolescents' access to parental support and care is blocked, and thus their core self-evaluation is impaired. Since previous studies have verified that a single risk factor could exert negative influences on core self-evaluation, it is more likely that cumulative risk factors could also have significant effects on core self-evaluation.

Beyond that, studies have also shown that core self-evaluation is significantly negatively correlated with problem behaviors. On the one hand, it has been found that core self-evaluation can significantly predict internalizing problem behaviors (e.g., anxiety and depression) (32). Hentrich et al. also found that core self-evaluation could be a significant shield from health-harmful outcomes, such as depression (33). On the other hand, researchers have also demonstrated a significant negative correlation between core self-evaluation and externalizing problem behaviors (e.g., aggression) (34, 35). For instance, Descartes et al. stated that the personal insecurities and self-loathing derived from low self-esteem (one of the manifestations of low core self-evaluation) could serve as an inner driver to trigger aggressive behaviors (34).

Based on the evidences above, and considering the relationship between cumulative ecological risk and core self-evaluation, as well as between core self-evaluation and problem behaviors, we proposed the following hypothesis:

**Hypothesis 3:** Core self-evaluation significantly mediates the relationship between cumulative ecological risk and problem behaviors.

### The Chain Mediation Effects

We also assumed that core self-evaluation is significantly correlated with basic psychological needs. First, having a high level of core self-evaluation often results in positive self-concepts (36), which makes them more confident in their decisions and gives them more autonomous control over their lives (satisfying their need for autonomy). Second, high core self-evaluation is often accompanied by a strong and solid sense of self-efficacy (37). Adolescents with high level of core self-evaluation are convinced of their competences to cope with immediate risks and more likely to take active approaches to defusing crises (satisfying their need for competence). Finally, when adolescents have high core self-evaluations, their emotional states tend to be positive and stable, which may shield them from negative affect such as anxiety and depression (32). Besides, they tend to show high social willingness and strong social skills which help them establish good social relations (satisfying their need for relatedness). In short, we infer that core self-evaluation positively predicts basic psychological need satisfaction. On the basis of these evidences and the other hypotheses (Hypothesis 2 and Hypothesis 3), we propose our last hypothesis:

**Hypothesis 4:** Cumulative ecological risk indirectly affects adolescents' problem behaviors through the chain mediating effects of core self-evaluation and basic psychological need satisfaction.

## Method

### Participants

Participants were 1,080 Chinese teenage participants (445 boys and 635 girls aged between 14 and 18, *M*_age_ = 16.76 years, *SD* = 1.19) who were selected from three secondary vocational schools in Jiangsu province based on cluster random sampling procedure. This study took a class as the unit to carry out a questionnaire survey. We delivered 1,100 questionnaires in total to participants at the beginning through the class teachers, and received 1,080 valid questionnaires, with the return rate of 98.2%.

This study was conducted after approval by the Institutional Review Board in the first author's university. Authorization was acquired from the secondary vocational schools' headmasters, giving us permission to carry out our investigation. Informed consent to participate in this study was provided by the participants' parents. The questionnaires required approximately 20 min to complete.

### Measures

#### Cumulative Ecological Risk

Based on the cumulative ecology theory (10, 38), this study comprehensively selected typical and representative risk factors in the four ecosystems of family, school, peer and community to construct the cumulative ecological risk index, which is in line with Cumulative Ecological Risk Questionnaire-Chinese Version (CERQ-CR) developed by Bao et al. (39). Specifically, the study included nine risk factors: lower parental education, more financial difficulties, worse parent-child relationship, worse parental relationship, less friend support, less school support, less neighborhood support, lower community security, and more negative life events.

#### Measures of Each Risk Factor

***Parental Education*. **This study referred to Gerald and Buehler (40) and used two items (e.g., “How far did your father/mother go in school?”) to investigate parental educational background respectively. Participants were required to respond from 1 (*never went to school*) to 7 (*master degree and above*). Lower scores in parental education reflected higher risks.

***Financial Difficulty*. **The Economic Strain Scale (ESS) developed by Wadsworth and Compas (41), translated and adapted by Wang et al. (42) was adopted to measure the participants' family financial difficulty. ESS covers four aspects, including food, clothing, housing and transportation. Each of them includes one item, for example, “We don't have enough money for new clothes” “My parents don't have enough money for the food I like to eat.” Items are measured on a five-point Likert scale ranging from 1 (*never*) to 5 (*always*). High scores reflect higher family financial risks. In this study, Cronbach's α coefficient of ESS was 0.89.

***Parent-Child Relationship*. **The parent-child relationship was evaluated with the cohesion subscale of Family Adaption and Cohesion Evaluation Scales II (43, 44). By adopting the 10-item scale, which ranges from 1 (*never*) to 5 (*always*), It was expected to assess how well the youth were getting along with their parents. Sample items include “My mother/father and I feel very close to each other,” “My mother/father and I are supportive of each other during difficult times.” Lower scores represent higher risks. Cronbach's α of this scale is 0.79.

***Parental Relationship*. **Parental relationship was assessed by a two-item scale (i.e., “Do your father and mother have a good relationship?”, “Do your father and mother quarrel?” reverse scored). The response format for these items was ranged from 1 (*very bad/never*) to 5 (*very good/always*). Lower scores on the items indicated worse parental relationship; in other words, higher risks. Overall internal consistency of the two-item scale was up to the standard (Cronbach's α = 0.70).

***Perceived Friend Support (PFS)*. **Perceived friend support was measured by using one sub-scale from the Multidimensional Scale of Perceived Social Support (45). PFS has four items (e.g., “My friends really try to help me”). The response format for PFS ranged from 1 (*strongly disagree*) to 7 (*strongly agree*). Lower scores reflected weaker perceived friend support and stronger risk. In this study, the reliability coefficient of PFS was 0.90.

***School Support*. **School support was assessed by six youth-report items (40). For each of the six items, teenagers should respond how true each statement is for themselves on a 5-point Likert scale (1 = *strongly disagree*, 5 = *strongly agree*). Sample items cover “I feel happy at school,” “I feel cared for by teachers.” Lower scores indicated less school support. In this study, Cronbach's α of school support scale was 0.95.

***Community Security*. **Community security was measured by one self-report item (40) that asked participant to rate the degree to which they felt safe living in their community on a four-point Likert scale from 1 (*strongly unsafe*) to 4 (*strongly safe*). Lower score manifested the unsafer community environment.

***Neighborhood Support*. **Referring to Bao et al. (39), we adopted two items to evaluate neighborhood support, such as, “Are you familiar with your neighbors?” on a four-point Likert scale from 1 (*no neighbors around*) to 4 (*very familiar*) and “When your family is in trouble, will your neighbors offer help?” on a four-point Likert scale from 1 (*no neighbors around*) to 4 (*always offer help*). Lower scores indicated less neighborhood support and more risks. Cronbach's α of neighborhood support scale was 0.70.

***Negative Life Event*. **We employed stressful life events scale (SLES) (46) to assess the negative experiences adolescents went through during the past year (e.g., “conflict or fighting against friends/classmates”). Responders indicated how accordant each statement is on a six-point Likert scale ranging from 0 = “*never happened*” to 5 = “*happened before and had a huge impact on me*.” The higher scores signify higher risks from negative life events. Cronbach's α of SLES was 0.89.

#### Constructing Cumulative Ecological Risk Index

Cumulative ecological risk index is constructed by dichotomizing each risk factor score (0 = no risk, 1 = risk) and then summing the dichotomous scores (10). Risk score assignment is accomplished by a statistical criterion (e.g., upper quartile of risk exposure = 1, all others = 0) (10, 40). Specifically, for the scales of parental education, parent-child relationship, community security, perceived friend support, school support, and neighborhood support, lower scores indicated more risks. Hence, scores below the 25th percentile in these scales were coded as one (*risk*) and the rest were coded with zero (*no risk*). However, for the scales of financial difficulty, parental relationship, and negative life event, higher scores indicated more risks. Therefore, scores above 75th percentile in these scales were assigned as one (*risk*), and the rest were coded with zero (*no risk*). Then, we added up all risk factor scores to obtain the final cumulative risk index ranging from 0 to 9. Higher cumulative risk index implies more severe cumulative risk.

#### Basic Psychological Need Satisfaction

We used the Basic Psychological Need Satisfaction Scale (BPNS) developed by Deci and Ryan (18). The Chinese version of BPNS translated by Yu et al. (47) was employed in the current study. The scale consists of 21 items to respectively measure the need for autonomy (seven items, e.g., “I generally feel free to express my ideas and opinions”), the need for relatedness (eight items, e.g., “I really like the people I interact with”), and the need for competence (six items, e.g., “Most days I feel a sense of accomplishment from what I do”). Participants were supposed to rate on how well three psychological needs were met in their real life from 1 (*strongly disagree*) to 7 (*strongly agree*). In this study, the Cronbach's α coefficients of total scale and three sub-scales were 0.90, 0.74, 0.82, and 0.71, respectively.

#### Core Self-Evaluation

We employed the Core Self-Evaluations Scale (CSES) developed by Judge et al. (28) to assess adolescents' fundamental self-evaluation. CSES comprises of 12 Likert-type items (1 = *strongly disagree and 5* = *strongly agree*). Subjects were supposed to estimate their agreement with items in the questionnaire. Sample item included “Overall, I am very satisfied with myself” and “Sometimes when I fail I feel worthless.” Higher scores reflected more positive and stronger core self-evaluations. In this study, the coefficient of Cronbach's α was 0.94.

#### Problem Behaviors

Problem behaviors consist of internalizing problem behaviors (e.g., anxiety and depression) and externalizing problem behaviors (e.g., aggression) (48). In the current study, the depression subscale and anxiety subscale of Irritability Depression and Anxiety Scale was applied to measure adolescents' internalizing problem behaviors (IDAS) (49). Each of subscales contained five items. Sample items included “I feel happy” from depression subscale (reverse scored), and “I feel nervous” from anxiety subscale. Participants appraised each item on a four-point Likert scale. In this study, both depression subscale and anxiety subscale were internally consistent, whose Cronbach's α coefficients were 0.75 and 0.73, respectively.

Besides, the Brief Version of Aggression Questionnaire (AQ, 12 items) was used to measure adolescent externalizing problem behaviors (50). Sample items included “I have threatened people I know,” and “At times I feel I have gotten a raw deal out of life.” Participants were asked to rate how much the statement applied to their real situation on a seven-point Likert scale (1 = *strongly disagree*, 7 = *strongly agree*). Cronbach's α of AQ was 0.91.

#### Control Variables

Previous research has noted that the variables of gender and age might be related to individuals' problem behaviors (51, 52). On that basis, we used these two variables as control variables in our hypothesis testing.

### Data Analysis

In this study, the structural model was used to test the mediating effects in this study by AMOS 22.0 software. First, we built several alternative models based on our hypotheses, and compared these models by using the following indices: χ^2^ fit index, Comparative Fit Index (CFI), Tucker-Lewis Fit Index (TLI), Root Mean Square Error of Approximation (RMSEA), and Standardized Root Mean Square Residual (SRMR). Fit values below 0.08 for RMSEA and SRMR, and fit values above 0.90 for CFI and TLI are generally considered to be a reasonable fit. The models were compared according to χ^2^ difference. Models with lower values of χ^2^ indicated a better fit (53). Meanwhile, the Akaike Information Criterion (AIC) was also used to evaluate models, with smaller values representing a better fit of the hypothesized model (54). Based on these fit indices, the best and final model was chosen. Then, we employed the bootstrapping procedure to test the significance of the multi-mediating effects in the final structure model. In this method, whether the 95% confidence interval of this effect value excluded zero determined the statistical significance of the mediating effect at *p* < 0.05 level (55).

## Results

### Correlation Analysis Among Variables

Table 1 rendered the means, standard deviations, and Pearson's correlations of all variables. Results manifested that the cumulative ecological risk was positively correlated with both internalizing problem behaviors (e.g., anxiety and depression) and externalizing problem behavior (e.g., aggression), while negatively related to core self-evaluation and basic psychological needs satisfaction. Core self-evaluation and basic psychological needs satisfaction were negatively correlated with two types of problem behaviors. Moreover, there was a significant positive correlation between core self-evaluation and basic psychological needs satisfaction (see Table 1).

**Table 1:** Correlation matrix for all variables.

**Variables**	**1**	**2**	**3**	**4**	**5**
1. Cumulative ecological risk	1.00				
2. Core self-evaluation	−0.34^***^	1.00			
3. Basic psychological needs satisfaction	−0.48^***^	0.64^***^	1.00		
4. Internalizing problem behavior	0.38^***^	−0.49^***^	−0.54^***^	1.00	
5. Externalizing problem behavior	0.34^***^	−0.38^***^	−0.39^***^	0.34^***^	1.00
Mean	2.94	3.22	4.45	2.00	2.72
SD	1.84	0.48	0.69	0.40	1.05

*** *p < 0.001*.

### Hypotheses Tests

**Figure 1:**
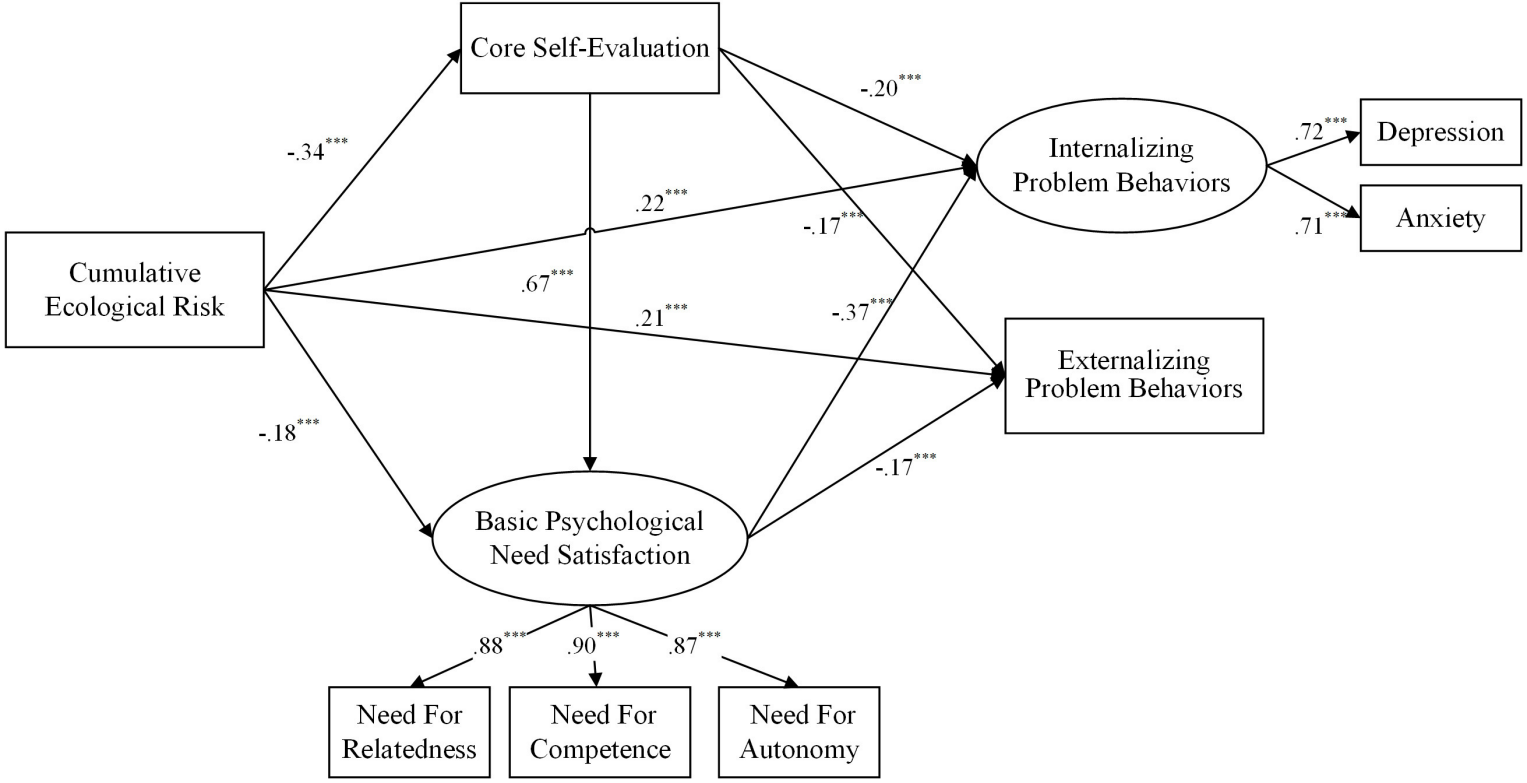
Final structural model with standardized estimated (Model 3). Need for relatedness, need for competence, need for autonomy, depression, and anxiety are dimensions of their corresponding latent variables. For the purpose of brevity, gender and age were included as control variables in M_3_ but not shown in the figure. ***p < 0.001.

To test the hypotheses, we built and compared three competitive models: a partial mediation model (M_1_), a full mediation model (M_2_), and a chain mediation model (M_3_). In these models, gender and age were all included as control variables with fixed effects on two types of problem behaviors. In M_1_, cumulative ecological risk and two types of problem behaviors were not only directly related but also indirectly related via the mediating variables while the pathways from cumulative ecological risk to two problem behaviors were constrained to zero in M_2_. The result indicated that a part of the fit indices of M_1_ or M_2_ were not satisfactory— M_1_: χ^2^(24) = 241.14, RMSEA = 0.09, SRMR = 0.04, CFI = 0.95, TLI = 0.91, AIC = 304.14; M_2_: χ^2^(26) =302.05, RMSEA = 0.10, SRMR = 0.05, CFI = 0.94, TLI = 0.89, AIC = 360.05. Comparing the fit of these two models, we found the model fit of M_2_ was significantly better than the fit of M_1_, Δχ^2^(2) = 60.91, *p* < 0.001. In order to find the best model, we constructed a chain mediation model (M_3_) in which we added the path from core self-evaluation to basic psychological needs satisfaction based on M_1_. The results showed an ideal fit to the data in M_3_, with χ^2^(23) = 180.02, CFI = 0.96, TLI = 0.93, RMSEA = 0.08, SRMR = 0.04, AIC = 244.02. Comparing the fit between M_3_ and M_1_, we found that M_3_ was better than M_1_, Δχ^2^(1) = 61.12, *p* < 0.001. Therefore, M_3_ was chosen as our final structural model (see Figure 1).

Then the Bootstrap estimation procedure was employed to test the significance of the effects in M_3_ (a bootstrap sample of 1,000 was specified). The results first showed that cumulative ecological risk positively predicted both internalizing problem behavior and externalizing problem behavior, supporting Hypothesis 1 (see Figure 1). Second, cumulative ecological risk was negatively related to both core self-evaluation (CSE) and basic psychological need satisfaction (BPNS). Third, both CSE and BPNS were negatively correlated with two types of problem behaviors (see Figure 1). Finally, a bootstrapping procedure revealed that both CSE and BPNS significantly mediated the relationship between cumulative ecological risk and two sorts of problem behaviors, and the significant mediation effects comprised: (1) the mediating effect of CSE between cumulative ecological risk and internalizing problem behaviors (β = 0.07, 95% CI: [0.03, 0.11]); (2) the mediating effect of BPNS between cumulative ecological risk and internalizing problem behaviors (β = 0.07, 95% CI: [0.04, 0.10]); (3) the mediating effect of CSE between cumulative ecological risk and externalizing problem behavior (β = 0.06, 95% CI: [0.02, 0.10]); (4) the mediating effect of BPNS between cumulative ecological risk and externalizing problem behavior (β = 0.03, 95% CI: [0.01, 0.06]); (5) the chain mediating effects of both CSE and BPNS between cumulative ecological risk and internalizing problem behaviors (β = 0.08, 95% CI: [0.06, 0.10]); (6) the chain mediating effects of both CSE and BPNS between cumulative ecological risk and externalizing problem behavior (β = 0.04, 95% CI: [0.02, 0.06]). Therefore, Hypotheses 2, 3, and 4 were supported.

## Discussion

Although researchers have drawn attention to the adverse consequences of cumulative ecological risk (14, 15), in-depth investigations of its internal mechanisms in causing problem behaviors are inadequate. This study put emphasis on the relationships of cumulative ecological risk with both internalizing behaviors (e.g., anxiety and depression) and externalizing behaviors (e.g., aggression), and especially the mediating mechanisms of core self-evaluation and basic psychological need satisfaction therein. To the knowledge of the authors, this is the first attempt to test the relative importance of core self-evaluation and basic psychological need satisfaction in the link between cumulative ecological risk and problem behaviors. Our results demonstrated that cumulative ecological risk was negatively related to internalizing and externalizing problem behaviors, which was in accordance with ecological system theory—risky social contexts could shape individuals' problem behaviors (56). Adolescents who were chronically exposed to cumulative ecological risk were more likely to engage in problem behaviors that hamper their normal lives, such as depression, anxiety, and aggression (17). This validated the cumulative risk model (10, 12).

The findings also uncovered the mediating effect of core self-evaluation. Specifically, our study revealed that cumulative ecological risk would reduce individuals' core self-evaluation, and it fosters problem behaviors in secondary vocational students. When facing multiple risk factors, secondary vocational students were too weak and incompetent to regulate and control the environments they live in. Consequently, it is easier for secondary vocational students to reduce their most basic evaluations of their ability and value, which means to reduce their core self-evaluations. Take it specific, individuals with low levels of core self-evaluation are inclined to underestimate or even deny their own coping abilities when facing risky situations, and finally get mired in negative feelings that would promote both internalizing and externalizing problem behaviors (33–35, 57).

Another finding of this research was that basic psychological need satisfaction could mediate the relationship between cumulative ecological risk and problem behaviors. First, cumulative ecological risk negatively predicted basic psychological need satisfaction. One possible explanation is that the cumulative risk factors basically mirror the shortage of supportive and irreplaceable growth resources essential to immature youths (12), so as to reduce psychological need satisfaction. Second, problem behaviors feed on unfulfilled psychological needs: failure to satisfy those needs triggers further problem behaviors. When their needs couldn't be satisfied for a long time, young people tended to engage in repeated problematic behaviors, such as bullying behaviors or internet addiction (24, 25), in order to attract others' attention or to express their appeals that were neglected. As recommended in previous studies (7, 58), depression and anxiety were selected as the indicators of internalizing problem behavior, while aggression serves as that of externalizing problem behavior. This study confirmed that the higher the cumulative ecological risk perceived by secondary vocational students, the more difficult it was to meet their basic psychological needs, and the more prone they were to involve in problem behaviors such as anxiety, depression, and aggression (26, 27).

In addition to the mediation mechanism expounded above, we also examined the chain mediating effect of core self-evaluation and basic psychological need satisfaction. As expected, the final model manifested that core self-evaluation mediated the relation between cumulative ecological risk and basic psychological need satisfaction, while basic psychological need satisfaction mediated the relation between core self-evaluation and internalizing and externalizing problem behavior. Cumulative ecological risk could significantly reduce the core self-evaluation of vocational students, and students with low core self-evaluation not only moved toward poor-ability and low-confidence self-cognition, but also preferred bad interactive tactics in interpersonal communication, such as avoidance (37). Thus, basic psychological needs (i.e., needs for autonomy, relatedness, and competence) were difficult to be satisfied. Ultimately, more internalizing and externalizing problem behaviors were likely to occur, forming a chained mediating path: “cumulative ecological risk → core self-evaluation → basic psychological need satisfaction → internalizing/externalizing problem behaviors.”

The outcome of this study has some important practical implications for precautions and interventions involving problem behaviors adolescents in vocational schools. First, secondary vocational students live in an environment that hides complex and diverse sources of risks. Family, school, and society should make every effort to create a positive living environment for vocational students so that they can avoid complex ecological risk factors. Educators ought to adopt integrated means to monitoring adolescents' growth environment and to identifying the high-risk groups of problem behaviors (14). Appropriate and targeted interventions was supposed to be implemented in time, so that teachers could detect which students may exhibit problem behaviors. Second, core self-evaluation and basic psychological need satisfaction mediated the link between cumulative ecological risk and problem behaviors. Therefore, a two-pronged approach should be adopted in intervention work, which attaches importance to the combined effects of core self-evaluation and basic psychological need satisfaction. On the one hand, effective interventions (e.g., narrative group counseling) should be taken to reduce the occurrence of problem behaviors by improving the students' core self-evaluations. On the other hand, families, schools, communities, and other organizations should offer more support to the students through a variety of measures, so as to improve their sense of competence, autonomy, and belonging, which will in turn reduce their incidence of problem behaviors.

Even though these results have both theoretical significance and practical implications, this study inevitably has some limitations. First, the nature of our study design was cross-sectional, which is difficult for drawing causality inferences. In the future, longitudinal or experimental studies could be adopted to investigate cause-and-effect relationships among cumulative ecological risk, core self-evaluation, basic psychological need satisfaction, and problem behaviors. Second, variables were assessed limited to self-report scale. Future study should draw on multiple sources of data (such as parents, peers, and teachers) to avoid common method bias as much as possible. Finally, this study was based on a sample of 1,080 Chinese secondary vocational students and thus it would be relatively difficult to extend the results to other age groups or occupational groups or to populations outside of China. Future research can verify the conclusions of this study among middle-aged adults who are exposed to more risks (e.g., midlife crisis), or further probe whether there are intercultural differences beyond cultural background.

## Data Availability Statement

The raw data supporting the conclusions of this article will be made available by the authors, without undue reservation.

## Ethics Statement

The studies involving human participants were reviewed and approved by Institutional Review Board in Jiangsu Normal University. Written informed consent to participate in this study was provided by the participants' legal guardian/next of kin.

## Author Contributions

PS designed, wrote, and approved all contributions to the study. YS participated in reviewing the literature and ran all analysis for the work. DF participated in collecting the data. HJ participated in designing the study. MP polished this article. All authors contributed to the article and approved the submitted version.

## Conflict of Interest

The authors declare that the research was conducted in the absence of any commercial or financial relationships that could be construed as a potential conflict of interest. Michele Smith is a junior review, in collaboration with LL.

## References

[1] Li L Huang C Chen X Tao L Huang Y Zhou X Reform course and development status of china's education system. In: Li L Zheng J Yu Z editors. Reform and Development of Educational System. Springer, Berlin, Heidelberg: Germany and Higher Education Press (2018). p. 23–231. 10.1007/978-3-662-55525-5

[2] Xu Y Chen X. Protection motivation theory and cigarette smoking among vocational high school students in China: a cusp catastrophe modeling analysis. Global Health Res Policy. (2016) 1:3–12. 10.1186/s41256-016-0004-9 29202053 PMC5675066

[3] Haug S Schaub MP Gross CS John U Meyer C Predictors of hazardous drinking, tobacco smoking and physical inactivity in vocational school students. Biomec Central Public Health. (2013) 13:475–84. 10.1186/1471-2458-13-475 PMC3658930 23672294

[4] Wang L The relationship of vocational students' psychological resilience, coping style and problem behavior. China J Health Psychol. (2017) 25:599–603. 10.13342/j.cnki.cjhp.2017.04.031

[5] Owens JS Hoza B. The role of inattention and hyperactivity/impulsivity in the positive illusory bias. J Consult Clin Psychol. (2003) 71:680–691. 10.1037/0022-006X.71.4.680 12924673

[6] Eisenberg N Cumberland A Spinrad TL Fabes RA Shepard SA Reiser M. The relations of regulation and emotionality to children's externalizing and internalizing problem behavior. Child Dev. (2001) 72: 1112–34. 10.1111/1467-8624.00337 11480937

[7] Colder CR Frndak S Lengua LJ Read JP Hawk LW Wieczorek WF. Internalizing and externalizing problem behavior: a test of a latent variable interaction predicting a two-part growth model of adolescent substance use. J Abnorm Child Psychol. (2017) 46:319–30. 10.1007/s10802-017-0277-6 28229368 PMC5568518

[8] Yeh KH Mediating effects of negative emotions in parent-child conflict on adolescent problem behavior. Asian J Soc Psychol. (2011) 14:236–45. 10.1111/j.1467-839X.2011.01350.x

[9] Ziv Y Sorongon A. Social information processing in preschool children: relations to sociodemographic risk and problem behavior. J Exp Child Psychol. (2011) 109:412–29. 10.1016/j.jecp.2011.02.009 21420102 PMC3096833

[10] Evans GW Li D Whipple SS Cumulative risk and child development. Psychol Bull. (2013) 139:1342–96. 10.1037/a0031808 23566018

[11] Appleyard K Egeland B Dulmen MHM Sroufe LA. When more is not better: the role of cumulative risk in child behavior outcomes. J Child Psychol Psychiatry. (2005) 46:235–45. 10.1111/j.1469-7610.2004.00351.x 15755300

[12] Lei H Zhang Q Li X Yang H Du W Shao J Cumulative risk and problem behaviors among Chinese left-behind children: a moderated mediation model. Sch Psychol Int. (2019) 40:1–20. 10.1177/0143034319835255

[13] MacKenzie MJ Kotch JB Lee LC Augsberger A Hutto N A cumulative ecological–transactional risk model of child maltreatment and behavioral outcomes: reconceptualizing early maltreatment report as risk factor. Child Youth Serv Rev. (2011) 33:2392–8. 10.1016/j.childyouth.2011.08.030 PMC4013824 24817777

[14] Li D Zhou Y Zhao L Wang Y Sun W Cumulative ecological risk and adolescent internet addiction: the mediating role of basic psychological need satisfaction and positive outcome expectancy. Acta Psychologica Sinica. (2016) 48:1519–37. 10.3724/SP.J.1041.2016.01519

[15] Ostaszewski K Zimmerman MA. The effects of cumulative risks and promotive factors on urban adolescent alcohol and other drug use: a longitudinal study of resiliency. Am J Community Psychol. (2006) 38:251–62. 10.1007/s10464-006-9076-x 17004127

[16] Stoddard SA Whiteside L Zimmerman MA Cunningham RM Chermack ST Walton MA. The relationship between cumulative risk and promotive factors and violent behavior among urban adolescents. Am J Community Psychol. (2013) 51:57–65. 10.1007/s10464-012-9541-7 22744013 PMC3684171

[17] Patwardhan I Mason WA Savolainen J Chmelka MB Miettunen J Järvelin MR. Childhood cumulative contextual risk and depression diagnosis among young adults: the mediating roles of adolescent alcohol use and perceived social support. J Adolesc. (2017) 60:16–26. 10.1016/j.adolescence.2017.07.008 28750265 PMC5684695

[18] Deci EL Ryan RM. The “what” and “why” of goal pursuits: Human needs and the self-determination of behavior. Psychol Inq. (2000) 11:227–68. 10.1207/S15327965PLI1104_0120204932

[19] Fraguela-Vale R Varela-Garrote L Carretero-García M Peralbo-Rubio EM. Basic psychological needs, physical self-concept, and physical activity among adolescents: autonomy in focus. Front Psychol. (2020) 11:491. 10.3389/fpsyg.2020.00491 32265796 PMC7100532

[20] González MG Swanson DP Lynch M Williams GC. Testing satisfaction of basic psychological needs as a mediator of the relationship between socioeconomic status and physical and mental health. J Health Psychol. (2014) 21:972–82. 10.1177/1359105314543962 25104782

[21] Taylor IM Lonsdale C. Cultural differences in the relationships among autonomy support, psychological need satisfaction, subjective vitality, and effort in British and Chinese physical education. J Sport Exercise Psychol. (2010) 32:655–73. 10.1123/jsep.32.5.655 20980709

[22] Vansteenkiste M Ryan RM On psychological growth and vulnerability: basic psychological need satisfaction and need frustration as a unifying principle. J Psychotherapy Integr. (2013) 23:263–80. 10.1037/a0032359

[23] Corrales T Waterford M Goodwin-Smith I Wood L Yourell T Ho C Childhood adversity, sense of belonging and psychosocial outcomes in emerging adulthood: a test of mediated pathways. Child Youth Serv Rev. (2016) 63:110–9. 10.1016/j.childyouth.2016.02.021

[24] Hein V Koka A Hagger MS. Relationships between perceived teachers' controlling behavior, psychological need thwarting, anger and bullying behavior in high-school students. J Adolesc. (2015) 42:103–14. 10.1016/j.adolescence.2015.04.003 25968108

[25] Yu C Li X Zhang W. Predicting adolescent problematic online game use from teacher autonomy support, basic psychological needs satisfaction, and school engagement: a 2-year longitudinal study. Cyberpsychol Behav Soc Netw. (2015) 18:228–33. 10.1089/cyber.2014.0385 25803769

[26] Yu C Li X Wang S Zhang W. Teacher autonomy support reduces adolescent anxiety and depression: an 18-month longitudinal study. J Adolesc. (2016) 49:115–23. 10.1016/j.adolescence.2016.03.001 27042976

[27] Wei M Shaffer PA Young SK Zakalik RA Adult attachment, shame, depression, and loneliness: the mediation role of basic psychological needs satisfaction. J Couns Psychol. (2005) 52:591–601. 10.1037/0022-0167.52.4.591

[28] Judge TA Erez A Bono JE Thoresen CJ The core self-evaluations scale: development of a measure. Pers Psychol. (2003) 56:303–31. 10.1111/j.1744-6570.2003.tb00152.x

[29] Hu J Wang Z Liden RC Sun J The influence of leader core self-evaluation on follower reports of transformational leadership. Leadersh Q. (2012) 23:860–8. 10.1016/j.leaqua.2012.05.004

[30] Song G Kong F Jin W Mediating effects of core self-evaluations on the relationship between social support and life satisfaction. Soc Indic Res. (2013). 114:1161–9. 10.1007/s11205-012-0195-5

[31] French KA Butts MM Allen TD Parent work conditions and adolescent core self-evaluations: examining the effects of work resource drain and parent gender. J Bus Psychol. (2015). 31:553–68. 10.1007/s10869-015-9429-2

[32] Hilbert A Braehler E Haeuser W Zenger M. Weight bias internalization, core self-evaluation, and health in overweight and obese persons. Obesity. (2013) 22:79–85. 10.1002/oby.20561 23836723

[33] Hentrich S Zimber A Sosnowsky-Waschek N Gregersen S Petermann F. The role of core self-evaluations in explaining depression and work engagement among managers. Curr Psychol. (2016) 36:516–30. 10.1007/s12144-016-9439-x

[34] Descartes CH Ramesar M Mills J Global or domain specific self-esteem: can it predict aggression among children and adolescents? J Aggression Maltreatment Trauma. (2018) 28:1–19. 10.1080/10926771.2018.1496960

[35] Valois RF Zullig KJ Revels AA Aggressive and Violent behavior and emotional self-efficacy: Is there a relationship for adolescents? J Sch Health. (2017) 87:269–77. 10.1111/josh.12493 28260243

[36] Lambert WW Lazarus RS Psychological stress and the coping process. Am J Psychol. (1970) 83:634–7. 10.2307/1420698

[37] Ferris DL Johnson RE Rosen CC Djurdjevic E Chang CH (Daisy), Tan JA. When is success not satisfying? Integrating regulatory focus and approach/avoidance motivation theories to explain the relation between core self-evaluation and job satisfaction. J Appl Psychol. (2013) 98:342–53. 10.1037/a0029776 22963514

[38] Bronfenbrenner U Morris PA The ecology of developmental processes. In: Lerner R editor. Handbook of Child Psychology. New York, NY: Wiley (1998). p. 993–1028.

[39] Bao Z Li D Zhang W Wang Y Sun W Zhao L Cumulative ecological risk and adolescents' academic and social competence: the compensatory and moderating effects of sense of responsibility to parents. Psychol Develop Educ. (2014) 30:482–95. 10.16187/j.cnki.issn1001-4918.2014.05.018

[40] Gerard JM Buehler C. Cumulative environmental risk and youth maladjustment: the role of youth attributes. Child Develop. (2004) 75:1832–49. 10.1111/j.1467-8624.2004.00820.x 15566383

[41] Wadsworth ME Compas BE Coping with family conflict and economic strain: the adolescent perspective. J Res Adolesc. (2002) 12:243–74. 10.1111/1532-7795.00033

[42] Wang J Li D Zhang W Adolescence's family financial difficulty and social adaptation: coping efficacy of compensatory, mediation and moderation effects. J Beijing Normal Univ. (2010) 22–32. 10.3969/j.issn.1002-0209.2010.04.002

[43] Olson DH Sprenkle DH Russell CS. Circumplex model of marital and family systems: I. cohesion and adaptability dimensions, family types, and clinical applications. Family Proc. (1979) 18:3–28. 10.1111/j.1545-5300.1979.00003.x 437067

[44] Fuligni AJ Zhang W. Attitudes toward family obligation among adolescents in contemporary urban and rural China. Child Develop. (2004) 75:180–92. 10.1111/j.1467-8624.2004.00662.x 15015683

[45] Zimet GD Dahlem NW Zimet SG Farley GK The multidimensional scale of perceived social support. J Pers Assess. (1988) 52:30–41. 10.1207/s15327752jpa5201_2 2280326

[46] Li D Zhang W Li X Li N Ye B. Gratitude and suicidal ideation and suicide attempts among Chinese Adolescents: direct, mediated, and moderated effects. J Adolesc. (2012) 35:55–66. 10.1016/j.adolescence.2011.06.005 21774977

[47] Yu C Zhang W Zeng Y Ye T Hu J Li D Gratitude, basic psychological needs, and problematic internet use in adolescence. Psychol Develop Educ. (2012) 28:83–90. 10.16187/j.cnki.issn1001-4918.2012.01.005

[48] Achenbach TM. The classification of children's psychiatric symptoms: a factor-analytic study. Psychol Monogr. (1966) 80:1–37. 10.1037/h0093906 5968338

[49] Snaith RP Constantopoulos AA Jardine MY McGuffin P. A clinical scale for the self-assessment of irritability. Br J Psychiatry. (1978) 132:164–71. 10.1192/bjp.132.2.164 623950

[50] Bryant FB Smith BD Refining the architecture of aggression: a measurement model for the Buss-Perry Aggression Questionnaire. J Res Pers. (2001) 35:138–67. 10.1006/jrpe.2000.2302

[51] Van der Ende J Verhulst FC Informant, gender and age differences in rating of adolescent problem behavior. Eur Child Adolesc Psychiatry. (2005) 14:117–26. 10.1007/s00787-005-0438-y 15959657

[52] Graves SL Blake J Kim ES Differences in parent and teacher ratings of preschool problem behavior in a national sample: the significance of gender and SES. J Early Intervention. (2012) 34:151–65. 10.1177/1053815112461833

[53] Marsh HW Hau KT Wen Z In search of golden rules: comment on hypothesis-testing approaches to setting cutoff values for fit indexes and dangers in overgeneralizing Hu and Bentler's (1999) findings. Struct Equation Model. (2004) 11:320–41. 10.1207/s15328007sem1103_2

[54] Akaike H Factor analysis and AIC. Psychometrika. (1987) 52:317–32. 10.1007/BF02294359

[55] Preacher KJ Hayes AF. Asymptotic and resampling strategies for assessing and comparing indirect effects in multiple mediator models. Behav Res Methods. (2008) 40:879–91. 10.3758/BRM.40.3.879 18697684

[56] Bronfenbrenner U Ecology of the family as a context for human development: research perspectives. Develop Psychol. (1986) 22:723–42. 10.1037/0012-1649.22.6.723

[57] Dou K Wang YJ Bin J Liu YZ Core self-evaluation, regulatory emotional self-efficacy, and depressive symptoms: testing two mediation models. Soc Behav Pers. (2016) 44:391–400. 10.2224/sbp.2016.44.3.391

[58] Sun P Sun Y Jiang H Jia R Li Z. Gratitude and problem behaviors in adolescents: the mediating roles of positive and negative coping styles. Front Psychol. (2019) 10:1547. 10.3389/fpsyg.2019.01547 31379645 PMC6646722

